# Secretory expression and purification of *Bacillus licheniformis* keratinase in insect cells

**DOI:** 10.1371/journal.pone.0183764

**Published:** 2017-08-23

**Authors:** Miaorong Huang, Ruiai Chen, Guangcai Ren

**Affiliations:** 1 Key Laboratory of Biotechnology and Drug Manufacture for Animal Epidemic Prevention, Ministry of Agriculture, Zhaoqing, China; 2 College of Veterinary Medicine, South China Agricultural University, Guangzhou, China; USDA-ARS, UNITED STATES

## Abstract

The keratinase (kerA) gene from *Bacillus licheniformis* PWD-1 was expressed and purified in insect cells. First, the sequence encoding Ker-His-Flag was designed based on the amino acid sequence of the protein and peptide and codon optimization in order to ensure the high expression in insect cells. In the next step, the synthetic DNA was inserted into the pUC57 vector and then sub-cloned in the pFastBac™-1 donor vector by *BamHI*/*HindIII* restriction sites. The constructed vector was transformed to *E*. *coli* DH10Bac™ cell to generate recombinant bacmid carrying Ker-His-Flag. Recombinant viruses were produced by infecting insect *Spodoptera frugiperda* (*Sf9*) cells with bacmid DNA and used for proteins production. Target proteins were purified from the cell supernatants by Ni^2+^-NTA affinity chromatography and evaluated by sodium dodecyl sulfate polyacrylamide gel electrophoresis (SDS-PAGE) and western blot. The purified product contained two peptides with molecular weights of 38 kDa and 30 kDa and had an optimal pH and temperature at 8.0 and 45°C for keratinolytic activity, respectively. The final product had a specific activity of about 635 U/mg. In summary, we have demonstrated that the open reading frame containing recombinant Ker-His-Flag was expressed and secreted by leader peptide of mellittin from *Apis mellitera* in insect cells and affinity purification through 8His-Flag tag. It presents an alternative technology for producing keratinases. To our knowledge, it was the first report on the expression of functional keratinase from *Bacillus licheniformis* in insect cells system.

## Introduction

Keratins are valuable protein sources for animal. However, keratins are insoluble proteins and cannot be digested by common proteases. They are components of a range of by-products occurring especially abundantly in slaughter houses and meat and poultry plants: skin remains, bristle, animal hair, horns and hooves, feathers, *etc* [1]. The structure of keratin is rich in disulfide bridges and sulfur compounds that make it insoluble and resistant to proteases lysis [2]. The traditional physical and chemical methods to processing keratins (high temperature, high pressure, acid or alkali treatment) not only loss important amino acids in keratins, consume a lot of energy, but also lead to serious environmental pollution[3]. Keratinases, a kind of metal or serine proteases, is used to designate the subset of proteases with keratinolytic activities. They are mainly produced by fungi, actinomycetes and bacteria, and decompose keratins into available amino acids and peptides. However, the low yield and the purification difficulties of wild-type strains restrict the industrial application of keratinases.

Recently, productions of keratinases using heterologous expression systems such as *Escherichia coli* [4–6], *Bacillus sp*. [7, 8] and *Pichiapastoris* [9, 10] have attracted considerable research interests. However, there are still several bottle necks in the production of recombinant keratinases in these expression systems, for instance, protein mal-folding as inactive inclusion body, lack of suitable or stable expression vectors, low secretion efficiency, *etc*. The insect cell baculovirus expression vector system (BEVS) is one of the most common eukaryotic expression systems. Compared to the other expression systems, it has a high level of intracellular expression with post-translational modification for exogenous gene. It has been used in many fields, including human medical research [11], influenza vaccines [12], and bioactive protein [13]. The current work aims to evaluate the insect cell baculovirus expression vector system as an alternative production technology for keratinases.

## Materials and methods

### Optimization and synthesis of recombinant protein coding sequence Ker-8His-Flag

In order to obtain a high expression level in insect, the coding sequence DNA was optimized based on the codon preference of the insect system by GenScript. Coding sequenceof recombinant protein was constructed by replacing the *Bacillus licheniformis* keratinase (GenBank No.: AAB34259.1) signal peptide coding sequencing with the signal peptide of mellittin from *Apis mellitera* (MKFLVNVALVFMVVYISYIYA) coding sequence, and adding the 8His-Flag tag (HHHHHHHHDYKDDDDK) coding sequence before stop codon. A *BamHI* restriction site with a Kozak sequence (GCCACC) and a *HindIII* restriction site were added to upstream and downstream of the hybrid kereatinase coding sequence, respectively. The final optimized DNA sequence containing *BamH*I site, sp (A. mellifera), truncated KerA (*Bacillus licheniformis*), 8His-Flag and *HindIII* site was synthesized by GenScript and cloned into pUC57 to generate cloning vector pUC-OKer-His-Flag.

#### Generation of recombinant baculovirus bacmid DNA carrying OKer-His-Flag

The 1.2 kb DNA sequence was excised from pUC-OKer-His-Flag and sub-cloned into the donor vector pFastBac™-1 (Life Technologies) by *BamHI*/*HindIII* restriction sites to create pFastBac1-OKer-His-Flag in which expression of recombinant protein is driven by polyhedrin promoter. Recombinant bacmid was generated by transforming pFastBac1-OKer-His-Flag into *E*. *coli* DH10Bac™ cells (Life Technologies). Badmic DNA was extracted using phenol/chloroform method and used for recombinant baculovirus production.

Transfer vector pFastBac1-OKer-His-Flag and bacmid DNA were confirmed by PCR and sequencing using the primer sets (pFastbac-F: TATTCCGGATTATTCATACC, pFastBac-R: ACAAATGTGGTATGGCTGA) and (MF13-F: GTTTCCCAGTCACGAC, MF13-R: CAGGAAACAGCTATGAC), respectively.

### Cell culture and recombinant virus rescue

*Spodoptera frugiperda* (*Sf9*, Invitrogen) cells were cultured in SF-900 medium (Gibco) with penicilin-streptomycin (100 μg/ml) and incubated at 27°C without CO_2_ exchange. Subconfluent cells were transfected with recombinant bacmid DNA using Cell fection transfection reagent (Invitrogen) following the manufacturer’s instructions. The cell culture supernatants were collected and centrifuged at 5000 rpm for 5 min to remove cell debris to obtain progeny 1 (P1) recombinant baculovirus 72 h post-transfection. P2 viruses were obtained through consecutive round of *Sf9* cells infection with P1 viruses. The supernatants (P2) were used to infect *Sf9* cells, thus generating P3 viruses. These viruses (P3) were used for recombinant protein production and analysis.

### Expression and purification of recombinant protein

Recombinant protein production was performed by infection of approximately 9×10^5^ *Sf9* cells with P3 recombinant viruses. The cell media were harvested 72 h post-infection and centrifuged at 5000 rpm for 20 min (at 4°C) to separate the supernatant from the pellet. The secreted protein in the supernatant was analyzed by sodium dodecyl sulfate polyacrylamide gel electrophoresis (SDS-PAGE), western blot and enzyme activity assay.

The supernatant was filtered through a 0.45 μm membrane after centrifugation at 12000 rpm for 10 min at 4°C, and followed by dialysis against 25 mM potassium phosphate (pH 8.0). The concentrated proteins were chromatographed on a nickel affinity column, which was pre-equilibrated with binding buffer (50 mM Tris, 500 mM NaCl, 0.1% Trion X-100, 1 mM DTT and 5% glycerol, pH 8.0). The column was then washed using wash buffer (50 mM Tris/HCl buffer, 500 mM NaCl, 0.1% Trion X-100, 1 mM DTT, 5% glycerol and 20 mM imidazole, pH 8.0). After that, the protein was eluted with elution buffer (250 mM imidazole, 300 mM NaCl, 10% glycerol and 300 mM imidazole, pH 8.0) and dialyzed against 20 mM phosphate buffer (pH 7.5). The resulting samples were concentrated to 2–3 mg/ml for further characterization or flash freezing in liquid nitrogen and stored at -80°C.

### SDS-PAGE and western blot analysis

The cell supernatants from *Sf9* cells infected with recombinant virus and the purified proteins were used as protein samples for SDS-PAGE and western blot analysis. Samples were prepared by mixing with 5× electrophoresis buffer, then heated at 95°C for 5 min. SDS-PAGE was performed using 10–12% separating polyacralaminde gel. After electrophoresis, gels were stained with Coomassie Brilliant blue R-250. For western blot analysis, protein samples were transferred to a polyvinylidene difluoride (PVDF) membrane after electrophoresis. Immune detection of OKer-His-Flag was carried out using the primary mouse monoclonal anti-FLAG (Sigma Cat. No. F1804) antibody and a secondary anti mouse horseradish peroxidase (HRP) conjugated IgG antibody. Then the reaction was developed by ECL kit (Amersham).

### Determination of keratinolytic activity

Keratinase activity was determined by measuring the azokeratin (Sigma) hydrolysis ability modified from the method of Lin, et al [14]. In short, the reaction mixture consisting of 1 ml diluted enzyme (about 0.1 mg/mL) and 1.5 mL substrate-phosphate solution (pH 7.5) was incubated at 37°C for 30 min, and then 1.0 mL 10% (w/v) trichloroacetic acid (TCA) was added to stop the reaction. After centrifugation at 10,000 ×g for 15 min, the absorbance of supernatant was measured at 450 nm. A control was performed by adding TCA to a reaction mixture before adding the enzyme solution. One unit of keratinase activity was defined as an increase in A_450_ of 0.01 under the required condition as described above in the test reaction compared with the control reaction.

### Effect of pH and temperature on keratinolytic actitity

The effect of pH on keratinase activity was determined by measuring the reaction of purified rKer at pH 5.0–11.0 (20 mM citrate buffer for pH 5.0–6.0, 20 mM Tris-HCl buffer for pH 6.0–9.0 and 20 mM glycine-NaOH buffer for pH 9.0–11.0) at 37°C, while the optimal temperature of purified rKer was measured from 30°C to 80°C at 5°C intervals with 20 mM sodium phosphate buffer (pH 8.0). All the reactions were performed as described in “Determination of keratinolytic activity”.

## Results

### DNA construction

**Fig 1:**
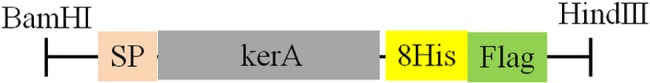
The structure of insert DNA OKer-His-Flag.

**Fig 2:**
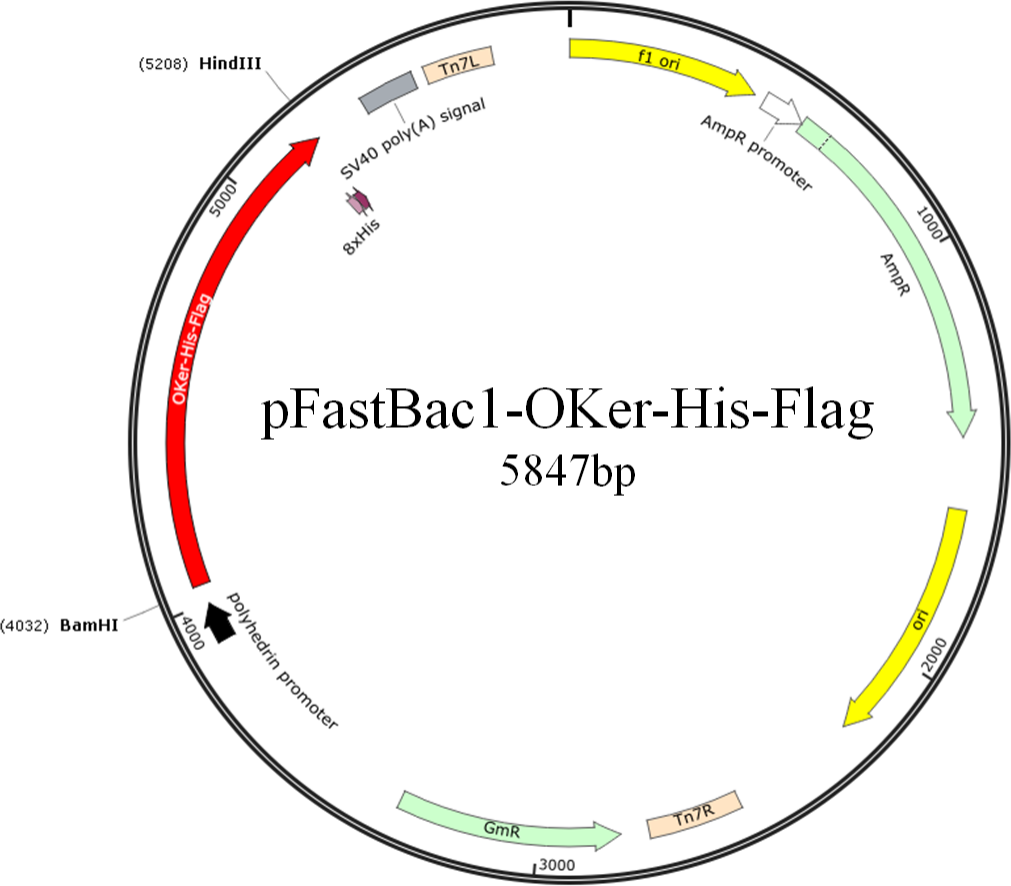
The graphic of transfer vector pFastBac1-OKer-His-Flag.

**Fig 3:**
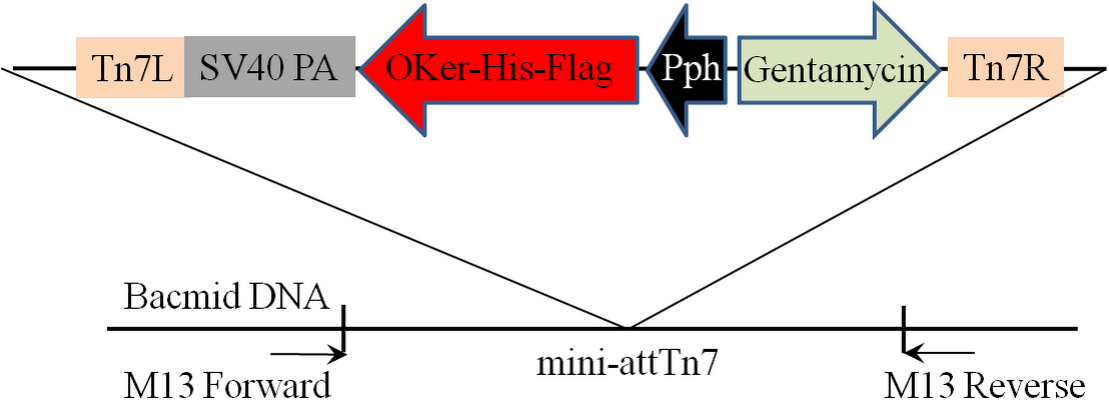
Map of recombinant bacmid DNA.

The 1.2 kb DNA sequence was ligated into pUC57 vector after codon optimization in insect system and chemical synthesis. The insert DNA (Fig 1) was digested from pUC-OKer-His-Flag and sub-cloned into pFastBac™-1vector through *BamHI*/*HindIII* restriction sites to generate transfer vector. The resulting plasmid, pFastBac1-OKer-His-Flag (Fig 2), contains all the necessary elements for bacterial homologous recombination: min Tn7 elements permit site-specific transposition of transgen into the bacculovirus genome when Tn7 transposition functions are provided in transform by a helper plasmid [15]; gentamicin resistance gene permits selection of recombinant bacmid in *E*. *coli* DH10Bac™ cells. Recombinant bacmid was obtained by transforming pFastBac1-OKer-His-Flag into *E*. *coli* DH10Bac™ cells. The recombinant bacmid contains a mini-F replicon, a kanamycin resistance marker, and attTn7. Expression cassettes, comprisinga strong AcMNPV polyhedrin promoter (P_PH_) which allows efficient, high-level expression of target gene in insect cells [16], is flanked by the left and right ends of Tn7. Fig 3 schematic represented the recombinant bacmid. All the constructions were confirmed by PCR and sequencing.

### Secretory and functional expression of recombinant protein in insect cell

**Fig 4:**
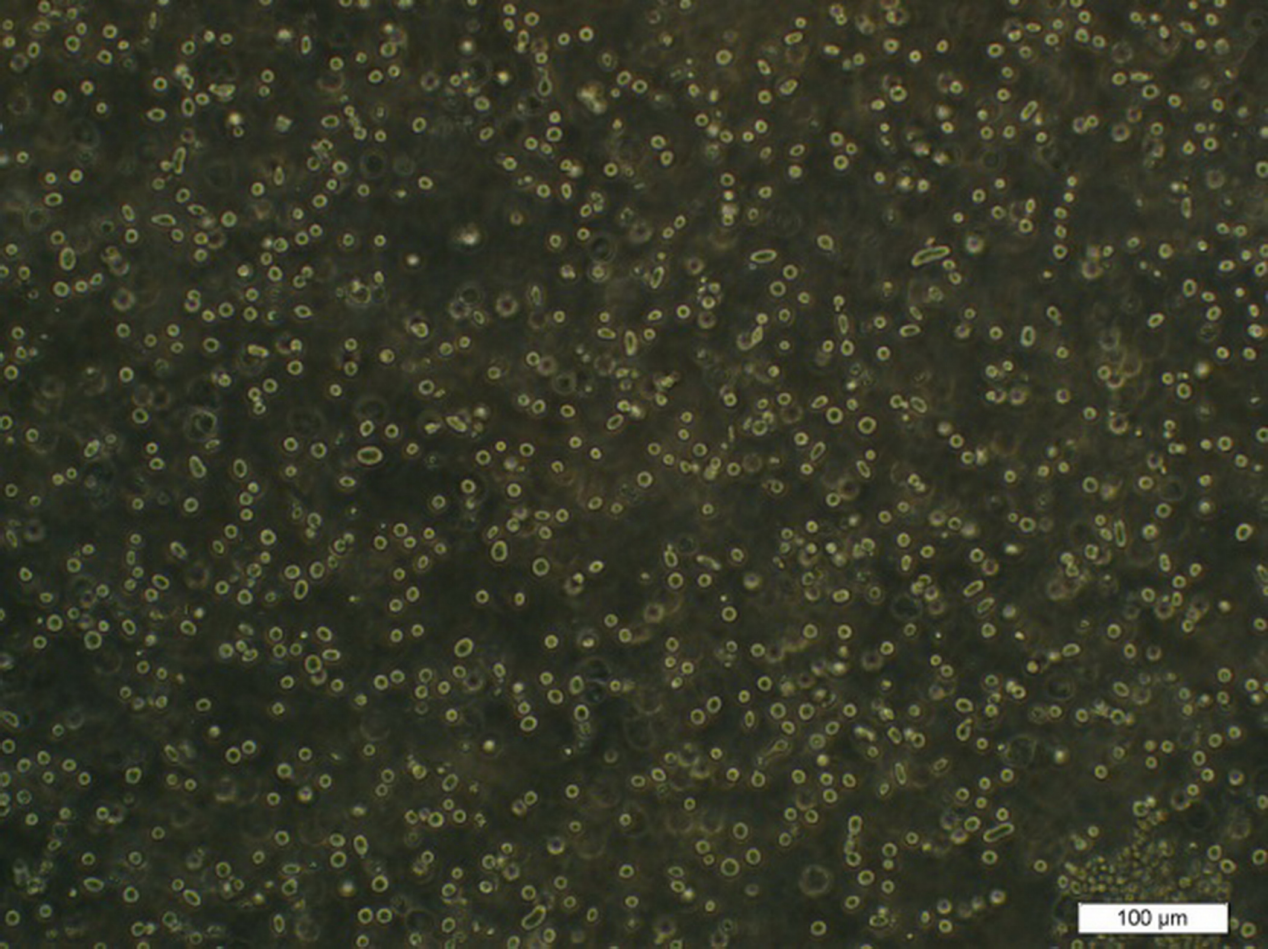
Cytopathic effects of *Sf9* cells infected with recombinant bacmid.

**Fig 5:**
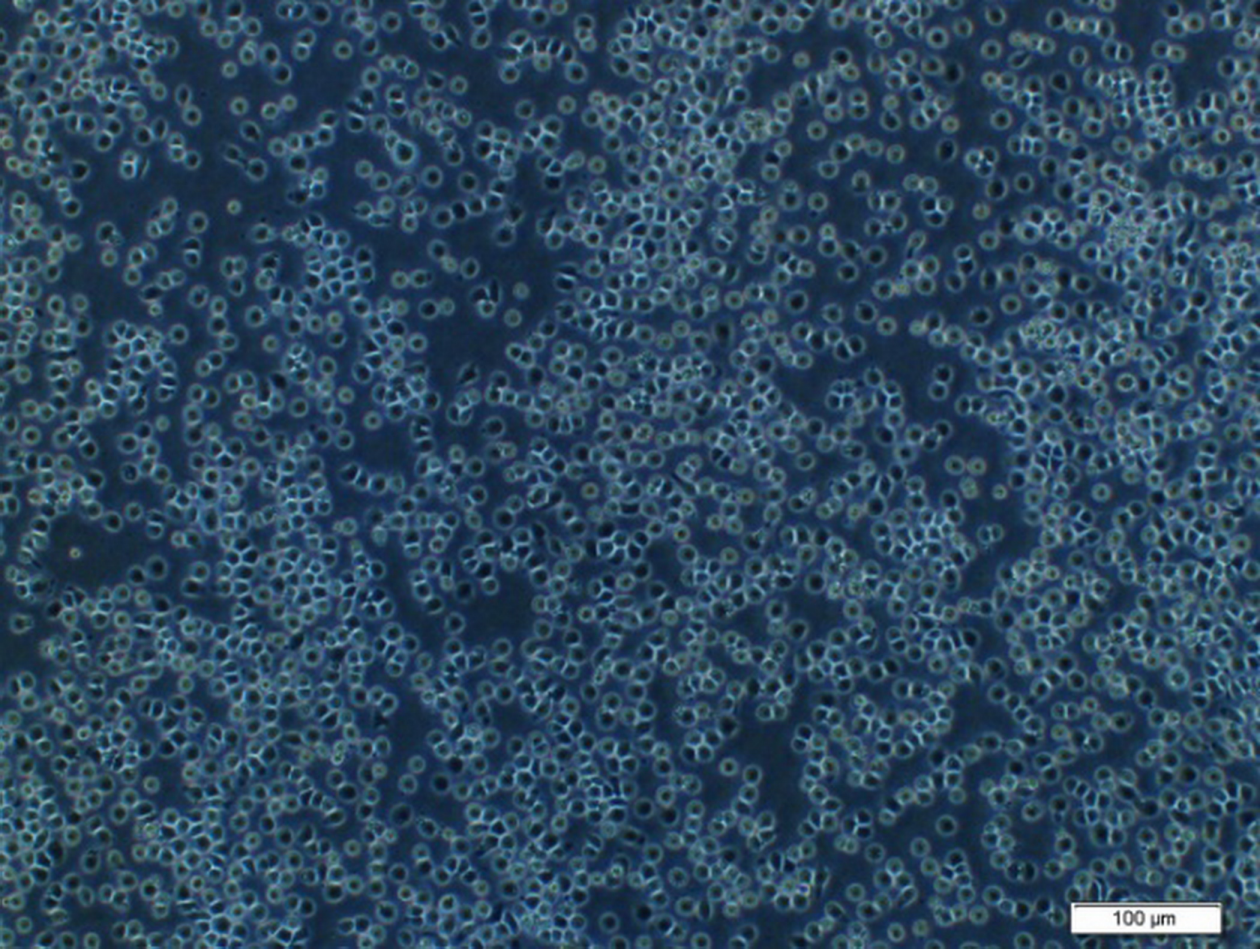
Un-infected cells.

Recombinant bacmid DNAs were isolated from DH10Bac and transfected into *Sf9* insect cells. Cytopathic effects (CPE) became apparent 72 hours post-infection. Cells became round, swollen, finally detached from the monolayer and die (Fig 4), comparing to the un-infected cells (Fig 5).

**Fig 6:**
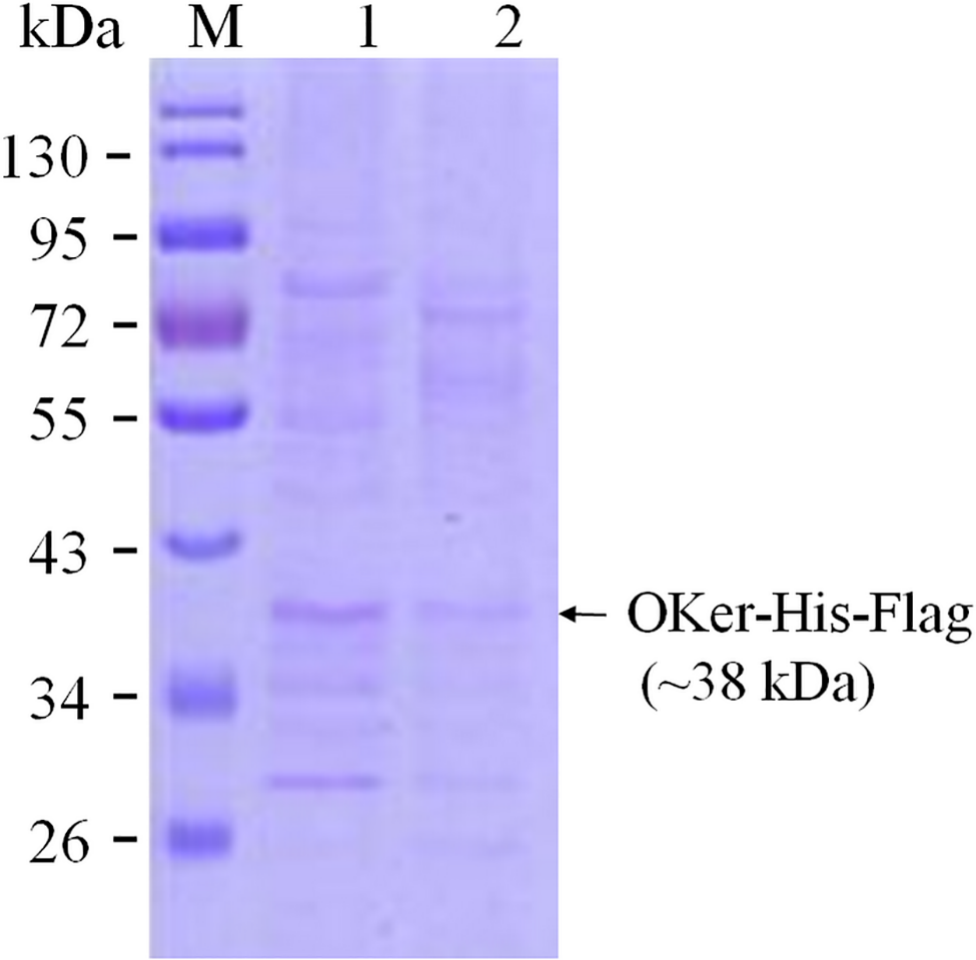
SDS-PAGE analysis of infected cell. Lane 1, cell supernatants and lane 2, cell lysates.

**Fig 7:**
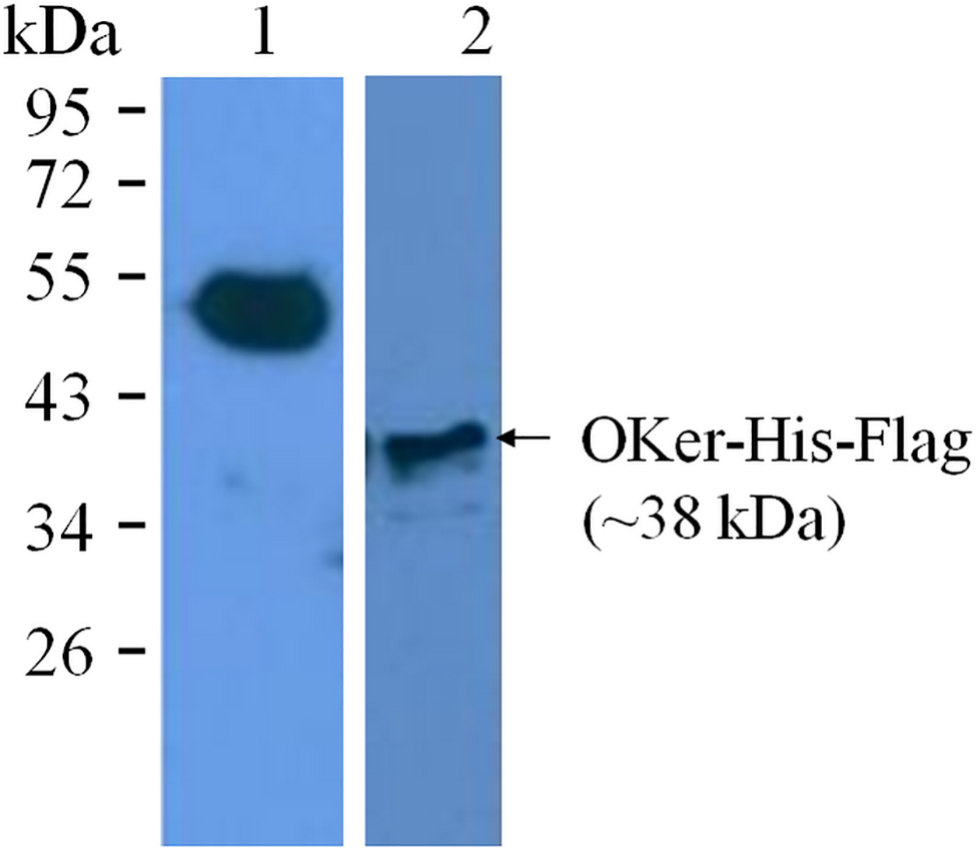
Western blot analysis. Lane 1, positive protein with Flag tag and lane 2, cell lysates. Signals of western blot were detected by a monoclonal antibody against C-terminus of Flag tag.

To assess the ability of the recombinant viruses to express the foreign gene, P3 recombinant viruses were used to infect *Sf9* cells. The cell lysates and cell supernatants were collected 72 hours post-infection and separated by SDS-PAGE. Western blot analysis was performed using mouse monoclonal anti-FLAG antibody as primary antibody. As showed in Fig 6 and Fig 7, a target band of a 38 kDa protein, which was almost similar to that of predicted (37.72 kDa), was confirmed in cell culture supernatants as well as in cell lysates, indicating that translation and secretion occurred in infected cells. There was no positive signal in uninfected cells, neither cell lysates nor cell supernatants. The results from culture supernatants indicated that the signal peptide of mellittin from *Apis mellitera* caused extra cellular accumulation of recombinant proteins in *Sf9* cells.

### Purification of recombinant protein

**Fig 8:**
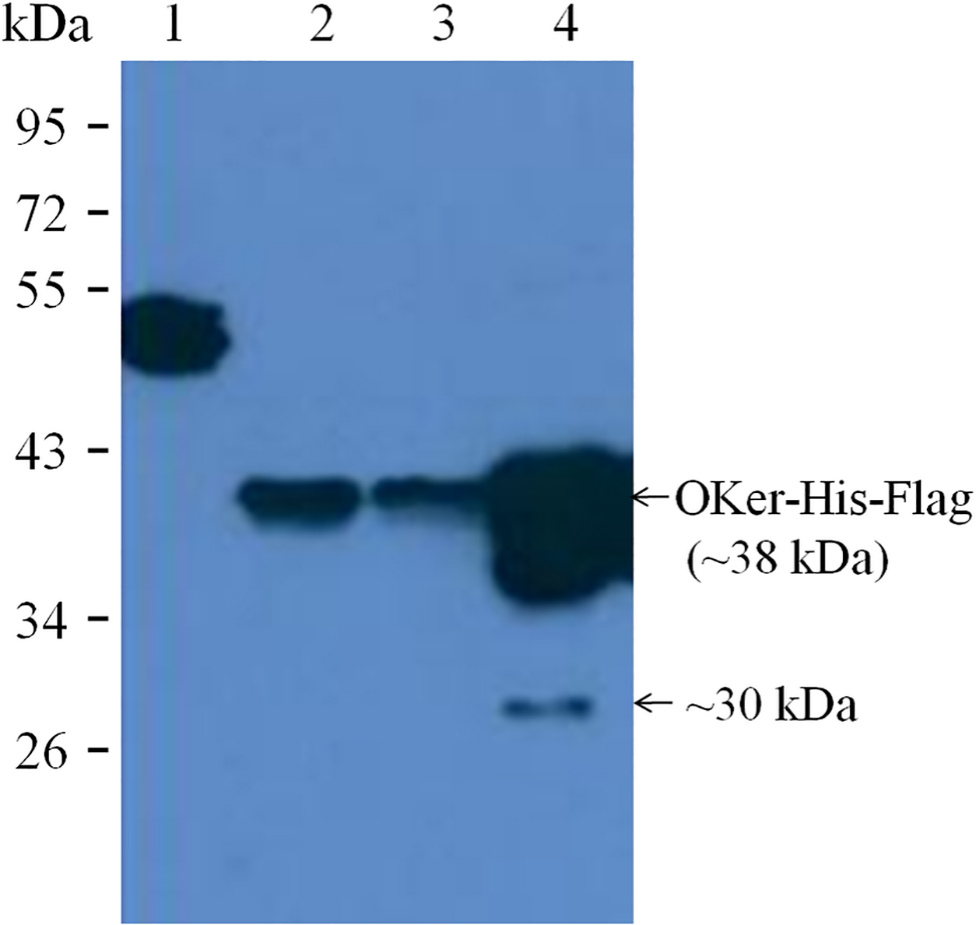
Western blot analysis on purified rKer eluted from the Ni^2+^-NTA affinity chromatography. Lane 1, positive protein with Flag tag; Lane 2, cell lysates; Lane 3, cell supernatants and lane 4, purity. Signals of western blot were detected by a monoclonal antibody against C-terminus of Flag tag.

Recombinant keratinase was purified from culture media by centrifugation, filtration, ammonium sulphate precipitation and nickel affinity chromatography. The overall purification factor was about seven-fold starting from the culture filtrate with a recovery of 24%. The final product had a specific activity of about 635 U/mg. This result illustrated that the expression of recombinant keratinase was successful and the enzyme was functional. The purity contained peptides with molecular weights of 38 kDa (most) and 30 kDa (a small fraction), estimated by western blot (Fig 8).

### Effect of pH and temperature on keratinase activity

**Fig 9:**
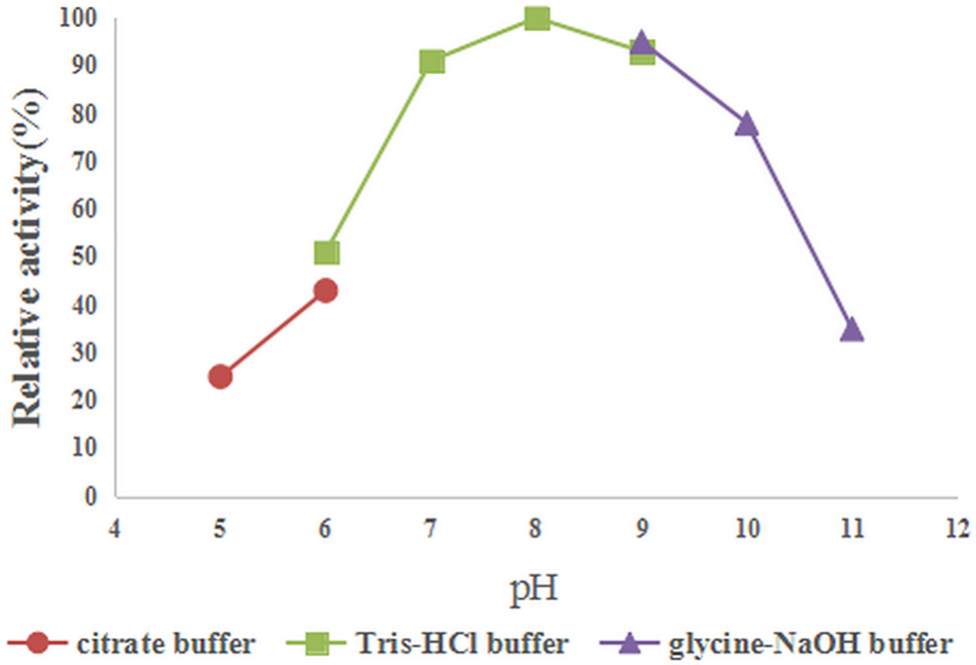
Effect of pH on keratinase activity.

**Fig 10:**
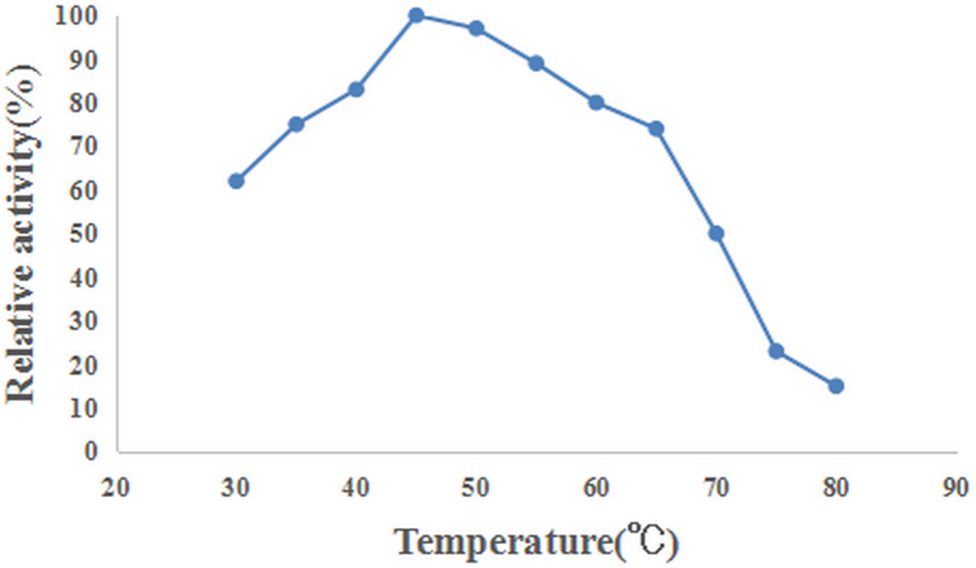
Effect of temperature on keratinase activity.

The pH optimum and apparent temperature optimum of keratinase activity were determined by the azokeratin hydrolysis assay. As shown in Fig 9, the enzyme was active at a broad range of pH values (pH 5.0 to 11.0). The optimal pH for keratinolytic activity was 8.0 and the enzyme activities declined rapidly at a pH higher than 10.0, but it was active even at pH 11.0, at 37°C. The optimal temperature for keratinase activity was 45°C, determined by varying the reaction temperatures, between 30°C and 80°C, at pH 8.0. Above 65°C, the activity sharply decreased (Fig 10). The enzyme exhibited 15% of the maximum activity at 80°C.

## Discussion

### Insect cells/ baculovirus system was suitable for expressing keratinases

Since keratin is an insoluble protein, the study and application of keratinases are of particular interest. Some researchers have been devoted to purifying and characterizing the newly-found enzymes from various microorganisms [14, 17–20], some searching for the suitable expression system for heterologous expression of keratinses [4, 6, 7, 10], and some others made efforts to improve the activity of the protease [21, 22]. For heterologous expression, *E*.*coli* has become the most commonly used system. However, accumulation as inactive inclusion bodies of the recombinant proteins is still an impediment. On the other hand, *Pichia* and *Bacillus* systems also present some technical challenges for industrial scale up. Insect cells such as *Sf9* and *High5* are the preferred expression hosts. To develop an alternative production and purification platform, the optimized coding sequence of rKer-His-Flag was inserted into the baculovirus vector to generate AcMPNV-Ker-His-Flag and expressed in *Sf9* cells. The recombinants were secreted into the cell cultural supernatants and purified by Ni^2+^-NTA affinity chromatography. The final product had a specific keratinolytic activity of about 635 U/mg. In comparison with the natural keratinase KerA purified from *Bacillus licheniformis* (6000 U/mg), weaker activity was observed for heterologous expression in insect cells of rKer. That could be as a result of the addition of purified tag 8His-Flag [23, 24]. However, the keratinalytic activity of recombinants from AcMPNV-Ker-His-Flag/Sf9 was higher than those of previous study using *E*.*coli* AD494(DE3) pLysS (12.0 U/mg)[6] and *B*.*subtilis* DB104 (143.1 U/mg) [7] as expression hosts transforming with rkeratinase from *Pseudomonas aeruginosa*, and also *Bacillus megaterium* (33.25 U/mg) [8] transforming with ker gene from *Bacillus licheniformis* MKU3, respectively.

### The suitable signal peptide for secretory expression

Signal peptide plays an important role in the secretory expression of gene expression system. The secretion efficiency of different signal peptides is varied. Therefore, the use of high efficient signal peptides is one of the important strategies to improve the yield of recombinant proteins [25]. In our report, the expression of recombinant proteins was conducted with self-signal peptide of *Bacillus licheniformis* keratinase or the signal peptide of mellittin from *Apis mellitera*. In the former method, the molecular weight of recombinant from cell lysates was about 43 kDa (S1 Fig), slightly larger than that of the later one (about 38 kDa). Furthermore, keratinase was undetected in culture supernatants from self-signal peptide recombinant, neither by immunoblotting analysis nor activity assays. These results indicated that the self-signal peptide of keratinase may not be cleaved in insect cells and that the heterologously expressed keratinases not be secreted into culture supernatants or correctly folded in insect cells. Meanwhile, the recombinant keratinases were secreted into cell culture supernatants in active forms by the signal peptide of mellittin from *Apis mellitera*, indicating that the signal peptide of mellittin from *Apis mellitera* functioned properly in insect cells and was required by the insect cells for an efficient secretion of the recombinant protein. Our results were consistent with previous reports that the suitable signal peptides are critically important for heterologous expression [5, 26–28].

### Heterogeneously processing of recombinants in insect cell expression systems

Immunoblot analysis of the rKer in cell cultural supernatants purified by Ni^2+^-NTA affinity chromatography revealed that the protein was largely secreted with a molecular mass of approximate 38 kDa, however, a minor fraction of 30 kDa was also observed (Fig 8 lane 4). Both forms can be recognized by monoclonal antibody against C-terminal FLAG tag, indicating that the 30 kDa form was generated from full-length peptide by removal of a portion of the N- terminal sequence. One possible explanation for the variable forms of the rKer might be that there are any sites of endo-proteolytic cleavage in the N-terminal of the protein. This kind of autolysis has also been reported by other scientists for various proteins expressed in the insect cell expression systems [29, 30]. The mechanism of the heterogeneously processing and whether it resulted in characteristic changes of the enzyme require future investigation.

### Characterization of the purified keratinase

The optimum pH of the purified recombinant keratinase activity, as determined by the azokeratin hydrolysis assay, was 8.0. It was not quite the same as the natural keratinase of *B*.*licheniformis* PWD-1, of which the optimum pH is 7.5[14]. However, the broad range of pH values (pH 5.0 to 11.0) of recombinant keratinases expressed in insect cells was in accordance with several previous reports that keratinases (express in wild-type and the recombinant strains) require working at neutral to high alkaline pH[9,31,32]. In addition, the recombinant keratinase was active at moderate temperature (30°C to 80°C) and still exhibited 15% of the maximum activity at 80°C. Our finding on the thermo active profile of keratinases is in line with other reports in various microorganisms [33–35].

## Conclusion

In summary, keratinase gene from *Bacillus licheniformis* was successfully expressed and secreted into the medium with keratinolytic activity by the insect cells/ baculovirus expression system. It was the first report on being able to constitute the functional keratinase from *Bacillus licheniformis* in insect cells system. Improvement of rKerA activity in this expression system is worthy of further study. The domain structure and functional amino acid residues of rKerA is also another topic for further studying the pH and thermal stabilities. These data will be helpful for further application of keratinases.

## Supporting information

S1 Fig Western blot analysis of recombinant proteins conducted with self-signal peptide of *Bacillus licheniformis* keratinase in cell lysates. Lane 1, positive protein with Flag tag; M, molecular weight standard; Lane 2, cell lysates. Signals of western blot were detected by a monoclonal antibody against C-terminus of Flag tag.
(TIF)
