# Supplementary figures and images for: Secretory expression and purification of Bacillus licheniformis keratinase in insect cells
Source: PLoS One. 2017 Aug 23;12(8):e0183764. doi: 10.1371/journal.pone.0183764 (PMC5568435; doi:10.1371/journal.pone.0183764)

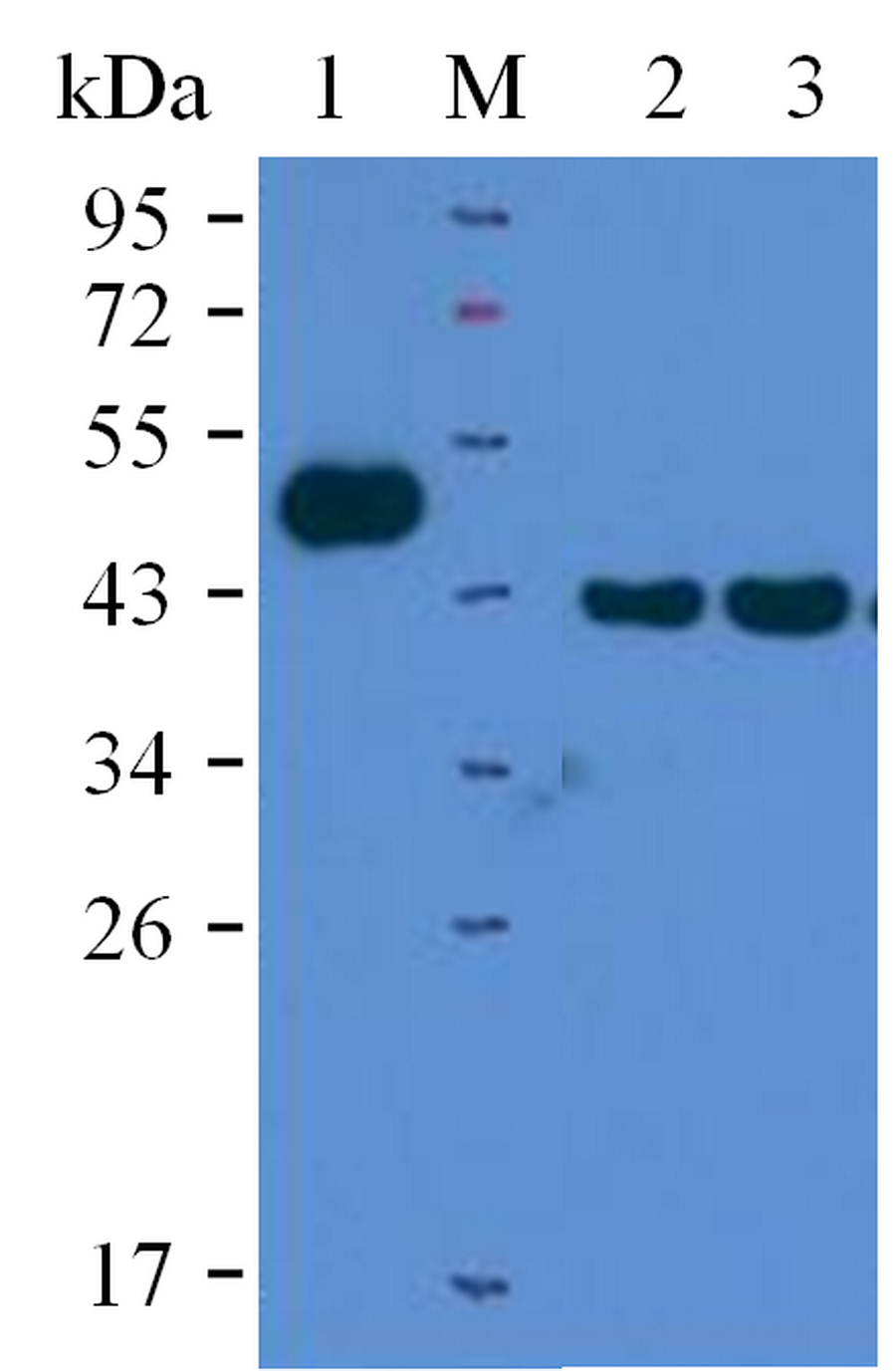

Supplement: S1 Fig — Lane 1, positive protein with Flag tag; M, molecular weight standard; Lane 2, cell lysates. Signals of western blot were detected by a monoclonal antibody against C-terminus of Flag tag. (TIF) [file pone.0183764.s001.tif]
